# The ameliorative effects of curcumin nanomicelle on testicular damage in the mouse model of multiple sclerosis

**DOI:** 10.1186/s12906-024-04423-3

**Published:** 2024-05-22

**Authors:** Farhad Koohpeyma, Zahra Khodaparast, Sara Salehi, Sina Danesh, Farhad Mohammadi Gheshlagh, Arzhang Naseri, Nima Montazeri-Najafabady

**Affiliations:** 1 Shiraz Endocrinology and Metabolism Research Center, Shiraz University of Medical Science, P.O. Box: 71345-1744, Shiraz, Iran; 2 Faculty of Veterinary Medicine, Ferdowsi University of Mashhad, Mashhad, Iran; 3 Trauma research center, Rajaei hospital, Shiraz University of Medical Sciences, Shiraz, Iran

**Keywords:** Multiple sclerosis, Curcumin, Nanoparticle, Testis, Antioxidant, Oxidative stress

## Abstract

**Background:**

This study investigated the effect of curcumin nanomicelle (CUR-n) on the structure of testis tissue, the process of spermatogenesis, LH, FSH, testosterone, and oxidative stress in a model of multiple sclerosis.

**Methods:**

Twenty-four male mice C57BL/6 were randomly allocated into 4 groups of 6 (1: group receiving 2% CPZ diet, 2: group receiving the diet of 2% CPZ + CUR-n with a dose of 50 mg/kg, 3: group receiving the diet of 2% CPZ + CUR-n with a dose of 100 mg/kg). The concentration of hormones (testosterone, LH and FSH), was measured by the special hormone assay ELISA kits. Measuring total antioxidant capacity (TAC) and Malondialdehyde (MDA) levels was done by spectrophotometry and calorimetric methods, respectively. Stereological analysis was done in order to explore the number of spermatogenesis cells, testis and sperm properties.

**Results:**

The results indicated that CUR-n (100 mg/kg) significantly enhanced the concentration of LH, FSH, testosterone, and TAC but reduced MDA levels. It also notably increased the quantity of spermatogonia, spermatocyte, round spermatids, long spermatids and LCs, augmented testis weight and volume, and germinal epithelium volume, improved sperm count, morphology, viability, and motility. In addition, a considerable decrease in the amount of wrinkling and disruption of the germinal epithelium was observed after intervention with CUR-n (100 mg/kg). Furthermore, a significant increase in the number of germ cells compared to the group receiving CPZ was detected.

**Conclusion:**

This study proposes that CUR-n could be a therapeutic agent for decreasing the adverse effects of MS on testis.

## Background

Multiple sclerosis (MS) is a complex disease of the brain and spinal cord in which immune cells attack myelinated axons. In this neurodegenerative disease, inflammation (caused by pro-inflammatory cytokines) and oxidative stress (triggered by reactive oxygen species (ROS)) induce demyelination, reduce remyelination, decrease axonal survival, and increase axonal damage together with massive activation of microglial cells [1, 2].

There is direct and mutual communication between MS and sexual dysfunction. The high prevalence of hypogonadism in males with MS and reduced serum testosterone levels in male mice with EAE has been reported [3].

Curcumin (CUR) or diferuloylmethane (1,7-bis(4-hydroxy-3-methoxyphenyl)-1,6-heptadiene-3,5-dione) [4] is a hydrophobic diphenolic extract from the rhizomes of *Curcuma longa* L. (turmeric) [5] with yellow color. It is a natural compound with antioxidant, anti-inflammatory, anticancer, antigrowth, anti-arthritic, anti-atherosclerotic, anti-depressant, anti-aging, anti-diabetic, anti-microbial, wound healing and memory‐enhancing activities [4].

The bottleneck in CUR research is its low availability in circulation and quick clearance which has hindered its generalized therapeutic application. Nanoparticle-based delivery systems have gained attention as a new strategy for enhancing the water solubility and bioavailability of therapeutic agents like CUR [6].

Previous studies have reported that CUR has properties that are related to the regulation of cell cycle regulatory proteins, enzymes, cytokines, and transcription factors in CNS-related disorders such as MS [7]. In addition, CUR-n has been identified as having high potential for the treatment of MS [8]. Motavaf et al., exhibited that dendrosomal CUR nanoparticles improved oligodendrogenesis and remyelination both in vitro and in vivo using models of MS demyelination [9]. Lu et al., [10] reported that CUR-loaded nanoparticles delay the progression of experimental autoimmune encephalomyelitis. Furthermore, previous in vivo studies showed the protective effects of CUR on reproductive system which related to anti-oxidant and anti-inflammatory features of CUR.

A randomized, double-blind, placebo-controlled clinical trial showed that curcumin supplementation could increase sperm quality, including total sperm count, sperm concentration and motility, and improved the total antioxidant capacity of plasma, malondialdehyde, C-reactive protein and tumor necrosis factor (TNF) [11]. Ranjbar et al., displayed that nano curcumin administration has significantly enhanced the sperm quality in AlP intoxicated rats [12]. Curcumin nanoparticles significantly improved oxidative status following ischemia-reperfusion injury in testicular tissues in rats [13]. The beneficial effects of curcumin nano-emulsion on spermatogenesis and reproductive performance in male rats under protein deficient diet model were also reported [14]. Moreover, nano-curcumin improved testicular function and sperm quality, especially in sperm motility and morphology in a rat model of varicocele [15].

Considering antioxidant, anti-inflammatory, anti-proliferative, and anti-differentiation, and neuroprotective properties of CUR we assumed in this study that its oral supplementation may protect testicular damage induced by CPZ in mice model of MS. According to our best information, the ameliorative effects of CUR nanoparticles on spermatogenesis and sexual hormones in CPZ-induced MS in male rats was not studied yet. So, this study was conducted to assess the effect of curcumin nanomicelle (CUR-n) on the structure of testis tissue, the process of spermatogenesis, LH, FSH, testosterone, and oxidative stress prompted by CPZ in a model of multiple sclerosis in male C57BL / 6 mice.

## Methods

### Animals

This study is an experimental animal intervention in which all protocols and ethical standards of working with animals are according to the guidelines of Shiraz University of Medical Sciences ethics committee. Twenty-four male mice C57BL/6 with an average weight of 23 g and an approximate age of 10 weeks, were included. Animals were selected randomly and taken to the animal room one week before the experiment began in order to adapt to the standard conditions. An animal room with a temperature of approximately 20 to 22 degrees Celsius and a light period of 12 h of lighting and 12 h of darkness and relative air humidity between 40% and 60% were provided for them. Mice were kept in a polycarbonate cage with a steel mesh roof. The mice’s bedding is made of sawdust and chips which was covered with wood. The floor of the cages will be changed and disinfected every week. All animal experiments comply with the ARRIVE guidelines and were carried out in accordance with the U.K. Animals (Scientific Procedures) Act, 1986 and associated guidelines, EU Directive 2010/63/EU for animal experiments.

### Curcumin nanomicelle (CUR-n)

CUR-n in the form of soft gel was purchased from Exir nano Sina company (SinaCUR®40, Iran). Each soft gel contains 40 mg of CUR surrounded by nanoparticles with a size of 10 nm.

### MS model induction and intervention groups

**Fig. 1:**
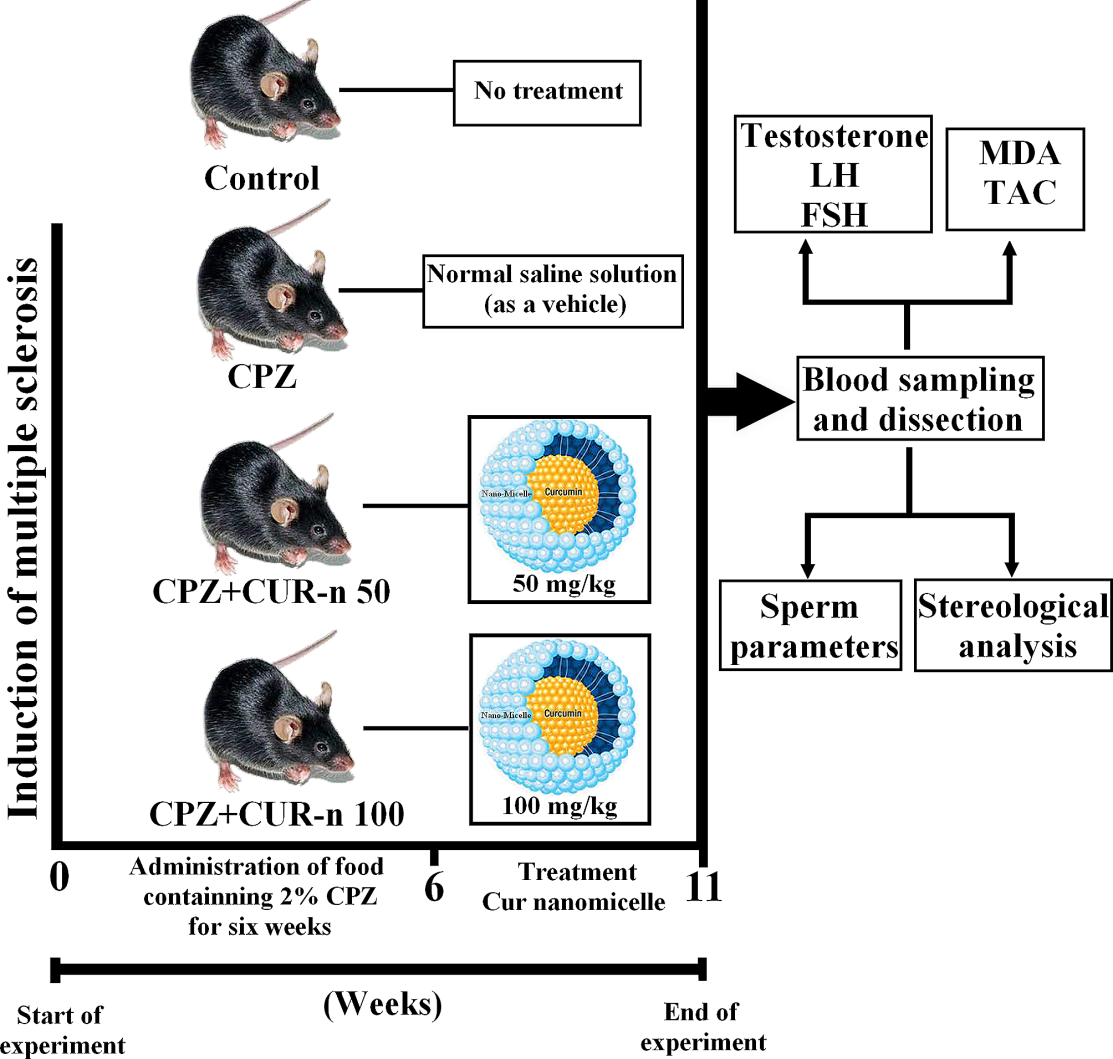
The scematic diagram of MS model induction and intervention groups

The schematic diagram of MS model induction and intervention groups was presented in Fig. 1. In order to create the MS model, first, the normal food of mice will be powdered by a mill. Then, a paste prepared from food containing 2% CPZ (Merck, Germany) was given to mice for six weeks in order to induce neurons injury and MS model.

In this study, mice were accidentally distributed into 4 groups of 6 as follows:

Control group: no treatment is done for the animals, and the animals only have water and normal food.

Experimental group 1: group receiving 2% CPZ diet for 35 days.

Experimental group 2: the group receiving the diet of 2% CPZ + CUR-n with a dose of 50 mg/kg for 35 days.

Experimental group 3: the group receiving the diet of 2% CPZ + CUR-n with a dose of 100 mg/kg for 35 days.

Administration of CUR-n was daily by needle gavage and oral. The experiment continued for 35 days, and the animals were weighed at the beginning and the end of the experiment. Finally, the animals were anesthetized with ketamine (80 mg/kg) and xylazine (5 mg/kg) mixture (Alfasn, Netherland, Co). At the end of the procedure, animals were euthanized according to the rules of the ethics committee of Shiraz University of Medical Sciences by placing them in a CO_2_ chamber. Rat blood samples were collected in sterile centrifuge tubes and were kept at room temperature for 20 min until it coagulates, then it was centrifuged for 15 min at a speed of 3000 rpm and its serum was separated.

On the other hand, after blood collection, the animals were dissected and their testicles were separated and washed with physiological serum and then placed in 10% formalin buffer solution for histopathological studies.

### Biochemical analysis

#### Measuring the concentration of sex hormones

To measure the hormone (testosterone, LH and FSH) levels, special hormone assay ELISA kits were used. For testosterone measurement, we used testosterone rat/mouse ELISA kit RUO (Catalog # MBS494055). FSH concentration was measured using the mouse Follicle Stimulating Hormone (FSH) ELISA kit (Catalog#FY-EM13638). Mouse Luteinizing Hormone (LH) ELISA Kit (Cat. No: MBS041300) was applied for LH measurement.

#### Total antioxidant (TAC) level measurement

Measuring TAC levels was done using the Spectrophotometry method under controlled temperature. In this method, ABTS (2-2-azino di–3-ethyl-Benzothiazoline sulfonate) incubates with peroxidase and H_2_O_2_ and produces cationic radical ABTS, which has a photo-absorption at 600 nm. Antioxidants in the sample inhibit color production and finally the amount of TAC will be reported in mmol/L.

#### Measuring the concentration of malondialdehyde (MDA)

The levels of free MDA, which is a byproduct and indicator of lipid peroxidation in cell membranes, were measured using thiobarbituric acid reactive substances (TBARS), following the method described by Zare et al. [16]. Briefly, the testes were homogenized in 1.15% KCl to create a 10% (w/v) homogenate. Subsequently, 0.9 ml of 1.8% sodium dodecyl sulphate (SDS), 1.5 ml of 20% acetic acid solution (pH = 3.5), and 1.5 ml of TBA solution were regularly added to 0.1 ml of tissue homogenates. The resulting homogenates were centrifuged at 4000 rpm for 10 min, and the MDA level was determined spectrophotometrically in the supernatant (λ = 532 nm).

### Stereological analysis

At first, the tissue of the left testicle was separated from all the waste tissues around it. Then, the weight of the testicle was calculated using a three-zero scale, and the initial volume (V1) was calculated using the flotation method. In this study, the Orientator method is used for cutting, so that the prepared cuts were Isotropic uniform random. In the next step, testicle pieces are placed in paraffin molds so that the trocar piece is placed in the middle of the other pieces. Then, sections with a thickness of 5 and 20 microns were made from each tissue by a microtome, and finally, hematoxylin-eosin (H&E) and Masson trichrome staining was done. After preparing the slides, the stereology software was used for the calculations, then the calculations were done as follows.

The amount of wrinkling is calculated based on the desired tissue volume using the following formula:

*Volume Shrinkage* = 1- (Area after/Area before)^1.5^.

The final tissue volume was obtained by multiplying volume shrinkage by the secondary volume.

Then, to calculate the volume ratio of the germinal epithelium, tubules, interstitial space, etc., the grid of cross points and the following formula were used:

$$Vv\left(structure\right)=\sum _{i=1}^{n}p \left(structure\right)/\sum _{i=1}^{n}\left(reference\right)$$

Calculation method to determine numerical density and absolute number of cells:

To do this, 20 micron sections, and the dissector technique, and the following formula were used:

$$Nv=\frac{\sum _{i=1}^{n}Q}{\sum _{i=1}^{n}P\times h\times \left(\frac{a}{f}\right)}\times \frac{t}{BA}$$

### Statistical analysis

At the end, in order to check the data in the desired groups, SPSS software version 22 was applied. One-way ANOVA and Tukey’s post hoc tests were done for group comparison.

## Results

### CUR-n significantly enhanced the concentration of LH, FSH, testosterone

**Fig. 2:**
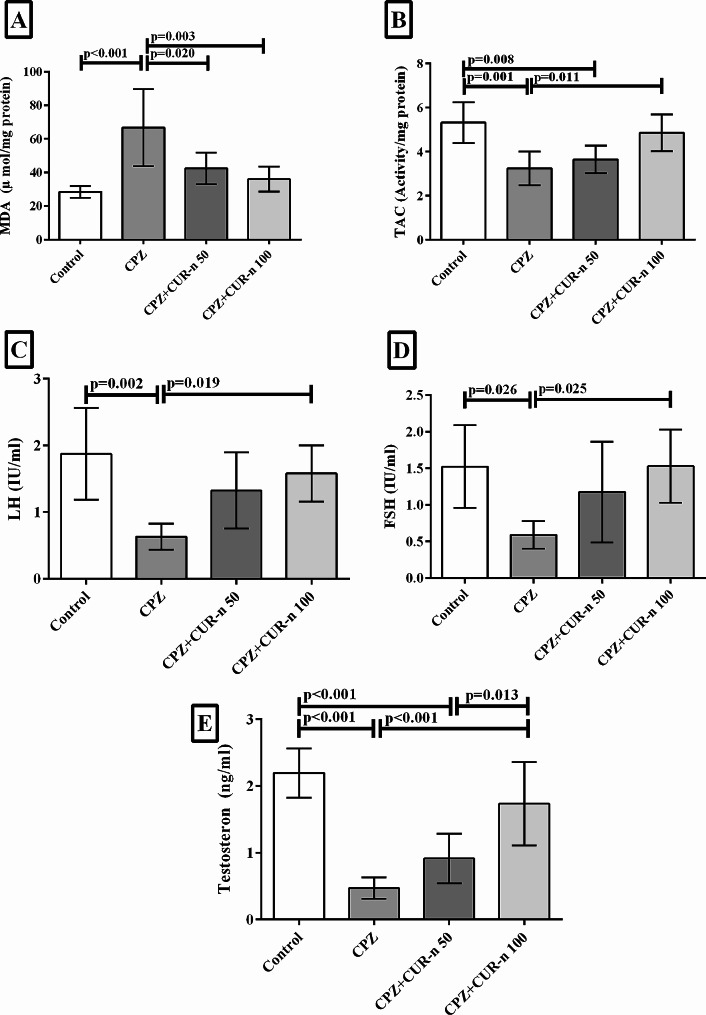
CUR-n effect on the levels of MDA (**A**), TAC (**B**), LH (**C**), FSH (**D**), testosterone (**E**)

CUR-n impact on the levels of LH, FSH, and testosterone was shown in Fig. 2. CPZ considerably, reduced the level of LH (*p* = 0.002), FSH (*p* = 0.026), testosterone (*p* < 0.001) compared to the control group. Intervention with CUR-n (100 mg/kg), significantly increased the level of LH (*p* = 0.019), FSH (*p* = 0.025), testosterone (*p* < 0.001). Besides, CUR-n (50 mg/kg) enhanced the level of LH, FSH, and testosterone but the changes were not significant. In regard to testosterone level, the difference between treatment with CUR-n **(**100 mg/kg) and (50 mg/kg) was significant (*p* = 0.013).

### CUR-n significantly improved antioxidant activity

In Fig. 2, we also presented the effects of CUR-n on TAC and MDA levels. CPZ notably, decreased TAC concentration (*p* = 0.001), and increased the level of MDA (*p* < 0.001) compared to the control group. Administration of CUR-n (100 mg/kg), significantly increased the level of TAC (*p* = 0.011), and decreased the level of MDA (*p* = 0.003). Also, CUR-n (50 mg/kg) enhanced the concentration of TAC, and decreased MDA level, but the changes were only significant in the case of MDA level (*p* = 0.02).

### CUR-n significantly increased spermatogonia, spermatocyte, round spermatids, long spermatids and LCs (LCs)

**Fig. 3:**
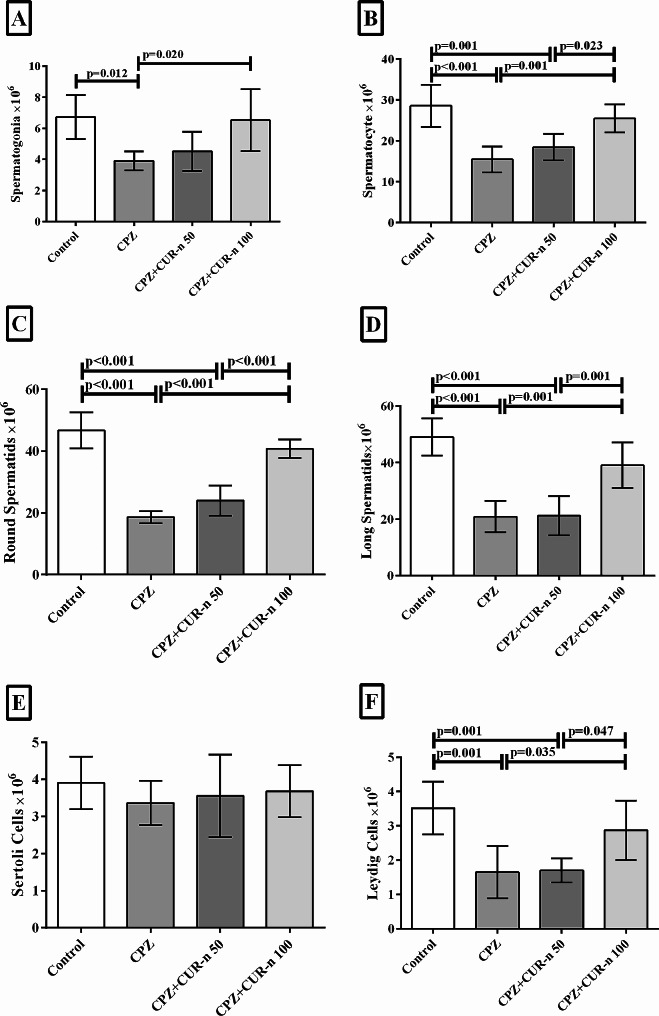
The effect of CUR-n on the number of spermatogonia (**A**), spermatocyte (**B**), round spermatids (**C**), long spermatids (**D**), sertoli (**E**) and LCs (**F**)

Figure 3 displays the effect of CUR-n on the number of spermatogonia, spermatocytes, round spermatids, long spermatids, sertoli and LCs. The MS induction by CPZ significantly decreased the number of spermatogonia (*p* = 0.012), spermatocyte (*p* < 0.001), round spermatids (*p* < 0.001), long spermatids (*p* < 0.001) and LCs (*p* = 0.001). Treatment with the CUR-n 100 mg/kg significantly increased the number of spermatogonia (*p* < 0.020), spermatocyte (*p* = 0.001), round spermatids (*p* < 0.001), long spermatids (*p* = 0.001), and LCs (*p* = 0.035) compared to the CPZ group. No significant changes in the number of spermatogonia, spermatocyte, round spermatids, long spermatids and LCs were observed after treatment with CUR-n 50 mg/kg. Also, significant variation in the case of spermatocyte (*p* = 0.023), round spermatids (*p* < 0.001), long spermatids (*p* = 0.001) and LCs (*p* = 0.047) was detected when CUR-n at the concentration of 50 mg/kg and 100 mg/kg compared with each other. CPZ and CUR-n showed no effects on the number of sertoli cells.

### CUR-n significantly augmented testis weight and volume, and germinal epithelium volume

**Fig. 4:**
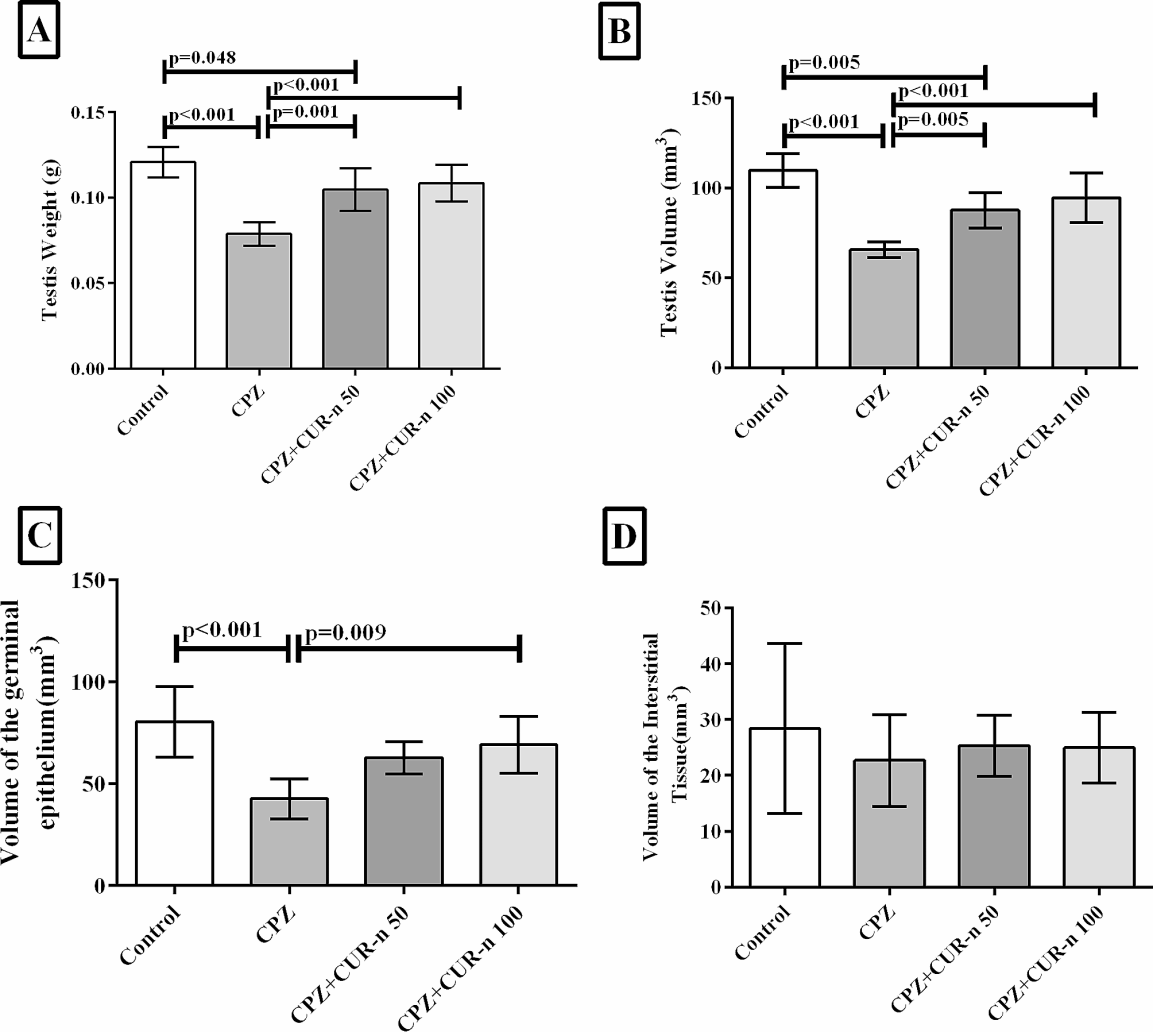
The effect of CUR-n on the testis weight (**A**) and volume (**B**), germinal epithelium volume (**C**) and intrestitial tissue volume (**D**)

As presented in Fig. 4, CPZ significantly reduced the weight and volume of the testis and also the volume of germinal epithelium. CUR-n (100 mg/kg) suppressed the destructive effects of CPZ after 35 days of treatment. CUR-n (100 mg/kg) significantly enriched the weight (*p* < 0.001) and volume (*p* < 0.001) of testis and the volume of germinal epithelium (*p* = 0.009). CPZ and CUR-n had no effects on the volume of interstitial tissue. In addition, no significant difference was found between CUR-n (100 mg/kg) and (50 mg/kg) respect to testis weight and volume, volume of germinal epithelium and interstitial tissue.

### CUR-n significantly increased sperm count and improved morphology and viability of sperm

**Fig. 5:**
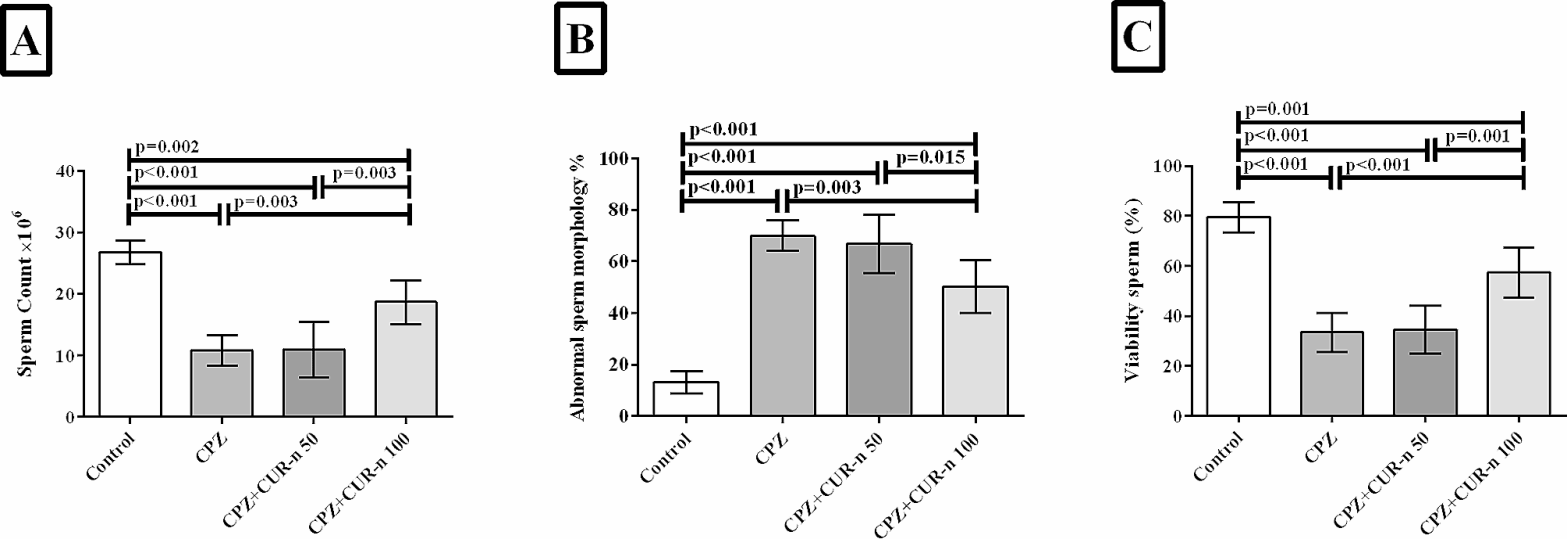
The effect of CUR-n on sperm count (**A**), morphology (**B**) and viability (**C**)

Figure 5 showed the ameliorative effect of CUR-n on sperm count, morphology and viability. After MS induction by CPZ, sperm count decreased (*p* < 0.001), the number of sperm with abnormal morphology increased (*p* < 0.001) and the number of viable sperm declined (*p* < 0.001). CUR (100 mg/kg) treatment reversed all these detrimental effects. CUR-n (100 mg/kg) significantly amplified the sperm count (*p* = 0.003), reduced the percentage of abnormal sperms (*p* = 0.003), and enhanced the number of viable sperms (*p* < 0.001). Also, the outcomes revealed a significant variation between treatment with CUR-n (100 mg/kg) and (50 mg/kg) in the case of sperm count (*p* = 0.003), morphology (*p* = 0.015) and viability (*p* = 0.001). No significant variation was observed after treatment with CUR-n (50 mg/kg) compared to CPZ group.

### CUR-n significantly retrieved the sperm motility

**Fig. 6:**
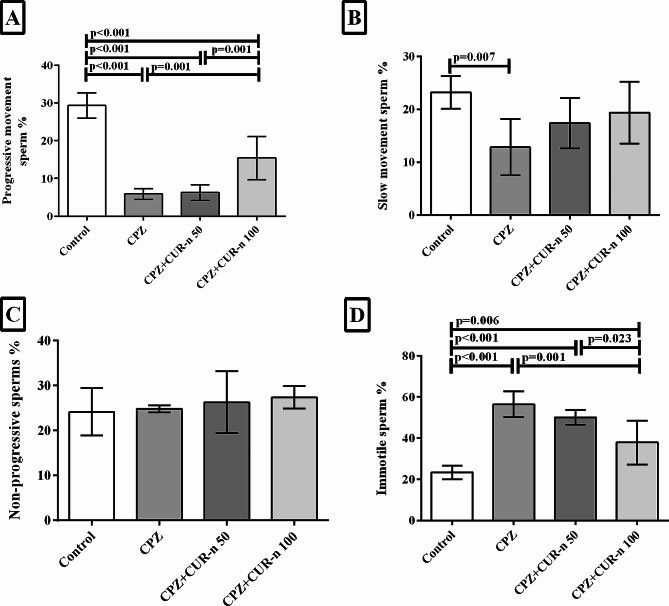
The effects of CUR-n on the percentage of progressive sperm (**A**), non-progressive sperm (**B**), sperm with slow movement (**C**), and immotile sperm (**D**)

The effects of CUR-n on the percentage of progressive sperm, non-progressive sperm, sperm with slow movement, and immotile sperm were existing in Fig. 6. CUR-n (100 mg/kg) meaningfully improved the percentage of progressive sperm and immotile sperm. CUR-n (100 mg/kg) had no effect on non-progressive sperms and sperms with slow movement. CUR-n at the concentration of (50 mg/kg), showed no ameliorative effects on progressive sperm, non-progressive sperm, sperm with slow movement, and immotile sperm.

### CUR-n impact on stereological parameters of testis tissue

**Fig. 7:**
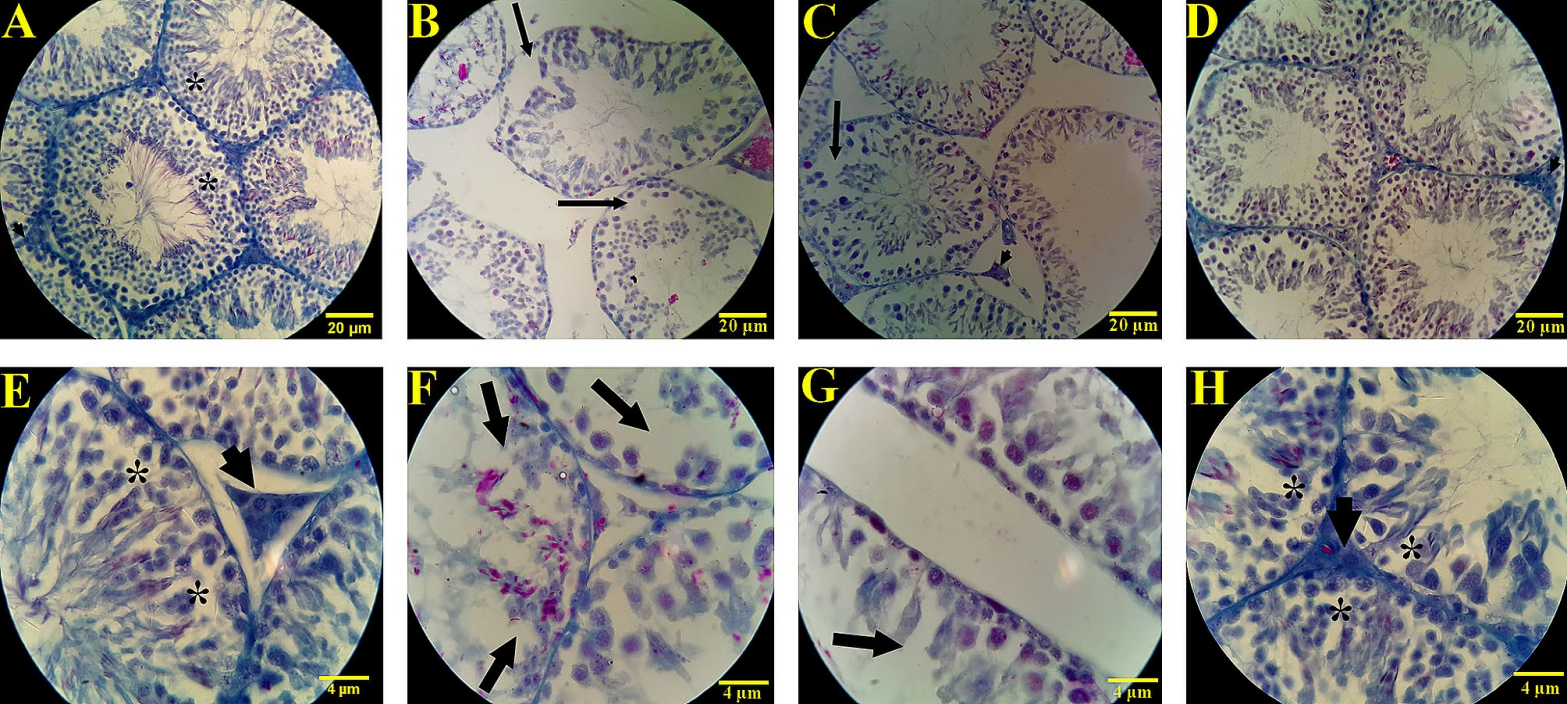
Histopathological changes of testicular tissue in healthy control groups (**A**, **E**), CPZ (**B**, **F**), CPZ + nanomicelle 50 mg/kg (**C**, **G**) and CPZ + nanomicelle 100 mg/kg (**D**, **H**). Mason’s trichrome staining, 400x. **A**, shows normal tubules and germ cells, full of spermatogonial cells, spermatocytes, spermatids, and spermatozoa, and no pathological changes were observed in the testicular tissue of this group. The asterisk indicates the normal process of spermatogenesis. In **B** and **C**, the tubules are wrinkled due to the disintegration of the germinal epithelium, the severe reduction in the number of germ cells and the presence of empty spots inside the tubules (arrow), and the pyknosis of the nucleus of Leydig cells show the destructive effects of CPZ on the testicular tissue. In **D**, which corresponds to the group receiving curcumin nanomicelles with a dose of 100 mg/kg, a significant decrease in the amount of wrinkling and disruption of the germinal epithelium was observed. Also, a significant increase in the number of germ cells compared to the group receiving CPZ was observed

Figure 7 displayed CUR-n effects on histopathological parameters of testis tissue. MS model induced shrinkage of the tubules, disintegration of the germinal epithelium, a sharp decrease in the number of germ cells, and LCs nucleus pyknosis. After intervention with CUR-n (100 mg/kg), a considerable decrease in the amount of wrinkling and disruption of the germinal epithelium was observed. Furthermore, a significant increase in the number of germ cells compared to the group receiving CPZ was detected.

## Discussion

The current work investigated the protective properties of CUR-n on oxidative stress, the process of spermatogenesis, LH, FSH, and testosterone levels, and histopathological parameters of testis tissue in the mouse model of MS. The findings indicated that CUR-n (100 mg/kg) significantly enhanced the concentration of LH, FSH, testosterone, and TAC but reduced MDA levels. Moreover, CUR-n significantly increased the number of spermatogonia, spermatocytes, round spermatids, long spermatids and LCs, augmented testis weight and volume, and germinal epithelium volume, improved sperm count, morphology, viability, and motility, and also retrieved histopathological parameters of the testis tissue.

CUR-n has been verified as an appropriate therapeutic agent for treatment of MS [8]. Mohajer et al., have designated neurotrophic properties of CUR and its nano-formulation, in a situation of CNS damage [17]. Naeimi et al., [18] demonstrated that CUR-charged nanoparticles enhance glial activation and increase myelin repair in the lyolecthine-induced focal demyelinating model of rat corpus calluses. Motavaf et al., established that nano CUR was active in improving the oligodendrogenesis of NSC progenitor cells and oligodendrocytes in a dose-dependent manner [9].

There is a strong direct link between hypogonadism and MS in males [19]. Milosevic et al., displayed that 40% of MS patients had declined testosterone concentrations [20]. In the mouse model of MS (EAE), Milosevic et al., found a reverse relationship between cytokine and testosterone levels, accompanied by an increase in serum luteinizing hormone (LH) levels, suggested that inflammatory cytokines suppress testosterone production by a direct effect on testicular LCs [20, 21]. In line with previously published articles, we observed that MS induction significantly declined testosterone levels.

The testosterone synthesis in males is mostly done by the LCs of the testis interstitium. Luteinizing hormone (LH) which is secreted from the pituitary gland in response to gonadotropin-releasing hormone (GnRH), generally regulate testosterone biosynthesis. LH direct impact on LCs is maintained by binding to a specific high-affinity receptor on LCs surface, the LH receptor (LHR). Binding of LH to LHR activates the cyclic adenosine monophosphate (cAMP)/protein kinase A signaling pathway [22].

The direct relationship between the levels of circulating gonadotropins (LH and FSH) and testosterone levels in the mouse model of MS was confirmed previously [20]. In this study, the results indicated that MS induction significantly reduced the serum levels of LH, FSH and testosterone which was in agreement with later studies [3, 23]. According to decrease in the number of LC in the MS model in this study, it seems that in addition to affecting the activity of LCs, MS has also affected LC numbers. Similarly, a previous study indicated that the dynamics of LC differentiation and proliferation and their functional capacity can be disrupted by MS induction [24]. Reduction of LH, FSH, and testosterone in the current study was representing for a damaged pituitary-testicular axis [25]. FSH and LH are important for the proliferation and normal function of Sertoli cells and Leydig cells, respectively. FSH is necessary for the proliferation, maturation, and function of Sertoli cells which generate signals for the initiation and maintenance of spermatogonia. Therefore, normal secretion of FSH is essential for maintaining spermatogenesis. LH induces the proliferation of Leydig cells and stimulates the secretion of testosterone which maintains the structural and functional integrity of reproductive organs and male accessory glands [26]. FSH and LH correspondingly interfere with spermatogenesis and steroidogenesis by affecting Sertoli and LCs [27]. In agreement with the results of our study, other investigations displayed the disruption of steroidogenesis pathway during MS which attributed to LH deficiency [23, 28]. Here, we also found that the number of the cells involved in spermatogenesis such as spermatogonia, spermatocyte, spermatids, Sertoli and LCs decreased after MS induction while, CUR-n (100 mg/kg) significantly reversed these adverse effects. Mohammdpour et al., confirmed that CUR can enrich the reproductive hormone (testosterone, FSH, and LH) levels in the CVS rats which might be due to the protective effect of CUR on LCs [29]. It could be explained that curcumin can inhibits Leydig cells apoptosis and thereby increases testosterone synthesis in testicular tissue [30].

In the current study, we observed that treatment with CUR-n significantly improved the sperm count, the percentage of sperm with normal morphology and the percentage of viable sperms. Similarly, Noorafshan et al., showed that CUR ameliorated the round or long spermatid, testis weight, volume, and germinal epithelium volume in metronidazole-treated rats [31]. Sadraei et al., reported that CUR-n 4 mg and 8 mg significantly increased sperm number and sperm motility and decreased sperm with abnormal morphology [15]. In consistent with our study, Abdelnour et al., demonstrated that treatment with nano-CUR at a concentration of 1.5 mg/ml reduced percentages of dead sperm, abnormalities, early apoptotic, apoptotic, and necrotic sperm cells [32].

In the current study, after CUR-n intervention serum level of MDA (an indicator of oxidative stress) decreased and TAC significantly increased. In line with our findings, Ahmed-Farid et al., exhibited that CUR nano-emulsion (2.5 and 5 mg/kg) enhanced serum testosterone levels, sperm motility, testicular GSH, CAT, SOD, testicular cell energy (ATP, ADP, and AMP), essential and non-essential amino acids in seminal plasma, and declined testicular MDA, NOx, GSSG and 8-OHDG [14]. Similarly, Sadraei et al., revealed that antioxidant therapy with nano CUR significantly reduced serum lipid peroxidation and ROS [15]. In a randomized clinical trial conducted by Alizadeh et al., CUR-n supplementation significantly improved total sperm count, sperm concentration, and motility, plasma levels of TAC, MDA, C-reactive protein, and tumor necrosis factor in comparison to the placebo [11]. CUR-n can reduce oxidative stress and increase antioxidant capacity which lead to improving sperm quality [11]. One possible explanation is that CUR prevents the production or propagation of free radicals in two ways: by competing with peroxidant metals at cell binding sites as a chelating agent and reducing the structural damage, and the other by improving the activity of antioxidant enzymes. The sulfhydryl groups in the structure of curcumin activate NRF2 (nuclear factor erythroid 2-related factor 2) as a transcription factor that expresses cytoprotective genes such as catalase (CAT), superoxide dismutase (SOD), and glutathione peroxidase (GPx) which leads to reduction of lipid peroxidation thereby improving sperm quality [33, 34].

Furthermore, the histopathological changes were investigated in this study. The results displayed that testis weight and volume, and germinal epithelium volume significantly decreased in CPZ-treated rats. CUR-n remarkably improved these changes and normalized the testicular tissue. Similar to our findings, Baharan et al., demonstrated that CUR could ameliorate germinal epithelium and interstitial tissue depletion, and also a reduction in the weight and volume of testis tissue in dianabol-treated mice [30].

The ameliorative effects of CUR-n on testicular damage in the mouse model of multiple sclerosis have not been directly addressed in the provided search results. The available articles discuss the effects of CUR-n in experimental models of multiple sclerosis, testicular toxicity in healthy rats induced by CUR-n, and the potential neuroprotective effect of CUR-n on learning and memory functions. This is the former study in which we explored the protective effects of CUR-n on sexual hormones, spermatogenesis cells, testis tissue histopathological properties in the mouse model of MS. This study suggests that CUR-n could be an appropriate candidate for declining the hostile effects of MS on male reproductive system but further in vivo and clinical studies showed be done to confirm the results of this study.

## Conclusion

To our best knowledge, this is the first study that investigated the ameliorative effects of curcumin nanomicelle on adverse effects of multiple sclerosis on male mice reproductive system. This study disclosed that curcumin nanomicelle could ameliorate oxidative stress, improve the sperm quality (especially, sperm count, motility, morphology, viability) and increase the number of spermatogenesis cells in multiple sclerosis model. Curcumin nanomicelle also enhanced LH, FSH, and testosterone levels. Curcumin nanomicelle considerably decreased the amount of wrinkling and disruption of germinal epithelium in testis tissue resulting from multiple sclerosis induction. Larger studies are needed to confirm the results of this investigation. Future studies should focus on more possible beneficial effects from using nano-curcumin on other male fertility parameters, and also inflammatory parameters and the advantageous consequences from using such nano curcumin on other multiple sclerosis complications on other tissues.

## Data Availability

The datasets generated during and/or analyzed during the current study are available from the corresponding author on reasonable request.

## References

[1] Theodosis-Nobelos P, Rekka EA. The multiple sclerosis Modulatory potential of Natural Multi-targeting antioxidants. Molecules. 2022;27(23). 10.3390/molecules27238402 PMC9740750 36500494

[2] Motavaf M, Sadeghizadeh M, Babashah S, Zare L, Javan M (2020). Protective effects of a Nano-Formulation of Curcumin against Cuprizone-Induced demyelination in the mouse Corpus Callosum. Iran J Pharm Res.

[3] Bove R, Musallam A, Healy BC, Raghavan K, Glanz BI, Bakshi R (2014). Low testosterone is associated with disability in men with multiple sclerosis. Mult Scler.

[4] Kunnumakkara AB, Bordoloi D, Padmavathi G, Monisha J, Roy NK, Prasad S (2017). Curcumin, the golden nutraceutical: multitargeting for multiple chronic diseases. Br J Pharmacol.

[5] Alibeiki F, Jafari N, Karimi M, Peeri Dogaheh H (2017). Potent anti-cancer effects of less polar curcumin analogues on gastric adenocarcinoma and esophageal squamous cell carcinoma cells. Sci Rep.

[6] Yavarpour-Bali H, Ghasemi-Kasman M, Pirzadeh M (2019). Curcumin-loaded nanoparticles: a novel therapeutic strategy in treatment of central nervous system disorders. Int J Nanomed.

[7] Monroy A, Lithgow GJ, Alavez S (2013). Curcumin and neurodegenerative diseases. BioFactors.

[8] Xie L, Li XK, Takahara S (2011). Curcumin has bright prospects for the treatment of multiple sclerosis. Int Immunopharmacol.

[9] Motavaf M, Sadeghizadeh M, Babashah S, Zare L, Javan M (2020). Dendrosomal nanocurcumin promotes remyelination through induction of oligodendrogenesis in experimental demyelination animal model. J Tissue Eng Regen Med.

[10] Lu L, Qi S, Chen Y, Luo H, Huang S, Yu X (2020). Targeted immunomodulation of inflammatory monocytes across the blood-brain barrier by curcumin-loaded nanoparticles delays the progression of experimental autoimmune encephalomyelitis. Biomaterials.

[11] Alizadeh F, Javadi M, Karami AA, Gholaminejad F, Kavianpour M, Haghighian HK (2018). Curcumin nanomicelle improves semen parameters, oxidative stress, inflammatory biomarkers, and reproductive hormones in infertile men: a randomized clinical trial. Phytother Res.

[12] Ranjbar A, Kheiripour N, Shateri H, Sameri A, Ghasemi H (2023). Protective effect of curcumin and Nanocurcumin on sperm parameters and oxidant-antioxidants system of Rat Testis in Aluminium Phosphide Subacute Poisoning. Pharm Nanotechnol.

[13] Esmaeilsani Z, Barati F, Mohammadi R, Shams-Esfandabadai N, Karimi I (2019). Effects of Curcumin nanoparticles on the tissue oxidative stress following testicular torsion and detorsion in Rat Model. Iran J Veterinary Surg.

[14] Ahmed-Farid OAH, Nasr M, Ahmed RF, Bakeer RM (2017). Beneficial effects of curcumin nano-emulsion on spermatogenesis and reproductive performance in male rats under protein deficient diet model: enhancement of sperm motility, conservancy of testicular tissue integrity, cell energy and seminal plasma amino acids content. J Biomed Sci.

[15] Sadraei MR, Tavalaee M, Forouzanfar M, Nasr-Esfahani MH (2022). Effect of curcumin, and nano-curcumin on sperm function in varicocele rat model. Andrologia.

[16] Zare S, Hossein Dabbaghmanesh M, Noorafshan A, Koohpeyma F, Bakhshayeshkaram M, Montazeri-Najafabady N (2019). Protective effect of vitamin E and vitamin C alone and in combination on testicular damage induced by sodium metabisulphite in rats: a stereological study. Andrologia.

[17] Mohajeri M, Sadeghizadeh M, Najafi F, Javan M (2015). Polymerized nano-curcumin attenuates neurological symptoms in EAE model of multiple sclerosis through down regulation of inflammatory and oxidative processes and enhancing neuroprotection and myelin repair. Neuropharmacology.

[18] Naeimi R, Safarpour F, Hashemian M, Tashakorian H, Ahmadian SR, Ashrafpour M (2018). Curcumin-loaded nanoparticles ameliorate glial activation and improve myelin repair in lyolecithin-induced focal demyelination model of rat corpus callosum. Neurosci Lett.

[19] Pakpoor J, Goldacre R, Schmierer K, Giovannoni G, Goldacre MJ (2014). Testicular hypofunction and multiple sclerosis risk: a record-linkage study. Ann Neurol.

[20] Milosevic A, Bjelobaba I, Bozic ID, Lavrnja I, Savic D, Tesovic K (2021). Testicular steroidogenesis is suppressed during experimental autoimmune encephalomyelitis in rats. Sci Rep.

[21] Dutta S, Sengupta P, Slama P, Roychoudhury S (2021). Oxidative stress, testicular inflammatory pathways, and male reproduction. Int J Mol Sci.

[22] Clément F, Yvinec R, Gallay N, Gagniac L, Guillou F, Crépieux P. The follicle-stimulating hormone signaling network in gonadal cells. Cellular Endocrinology in Health and Disease: Elsevier; 2021. p. 421 – 43.

[23] Milosevic A, Janjic MM, Lavrnja I, Savic D, Bozic ID, Tesovic K (2020). The sex-specific patterns of changes in hypothalamic-pituitary-gonadal axis during experimental autoimmune encephalomyelitis. Brain Behav Immun.

[24] Ivell R, Wade JD, Anand-Ivell R (2013). INSL3 as a biomarker of Leydig cell functionality. Biol Reprod.

[25] Turner TT, Lysiak JJ (2008). Oxidative stress: a common factor in testicular dysfunction. J Androl.

[26] Oduwole OO, Peltoketo H, Huhtaniemi IT. Role of follicle-stimulating hormone in spermatogenesis. Front Endocrinol. 2018;9. 10.3389/fendo.2018.00763 PMC6302021 30619093

[27] Priya PH, Girish BP, Reddy PS (2014). Restraint stress exacerbates alcohol-induced reproductive toxicity in male rats. Alcohol (Fayetteville NY).

[28] Selvaraj V, Stocco DM, Clark BJ (2018). Current knowledge on the acute regulation of steroidogenesis. Biol Reprod.

[29] Mohamadpour M, Noorafshan A, Karbalay-Doust S, Talaei-Khozani T, Aliabadi E (2017). Protective effects of curcumin co-treatment in rats with establishing chronic variable stress on testis and reproductive hormones. Int J Reproductive Biomed.

[30] Baharan O, Goodarzi N (2018). Protective effects of Curcumin on the Structural Parameters of Seminiferous Tubules and Leydig cells in Methandienone Treated Mice. Res J Pharmacognosy.

[31] Noorafshan A, Karbalay-Doust S, Valizadeh A, Aliabadi E, Mirkhani H. Ameliorative effects of Curcumin on the Seminiferous Epithelium in Metronidazole-treated mice:a Stereological Study. 2010;38(3):366–71. 10.1177/0192623310362248 20215582

[32] Abdelnour SA, Hassan MAE, Mohammed AK, Alhimaidi AR, Al-Gabri N, Al-Khaldi KO et al. The Effect of adding different levels of Curcumin and its nanoparticles to Extender on Post-thaw Quality of Cryopreserved rabbit sperm. Anim (Basel). 2020;10(9). 10.3390/ani10091508 PMC7552309 32858961

[33] Abdelnour SA, Hassan MAE, Mohammed AK, Alhimaidi AR, Al-Gabri N, Al-Khaldi KO et al. The Effect of adding different levels of Curcumin and its nanoparticles to Extender on Post-thaw Quality of Cryopreserved rabbit sperm. Anim [Internet]. 2020; 10(9). 10.3390/ani10091508 PMC7552309 32858961

[34] Santonastaso M, Mottola F, Iovine C, Colacurci N, Rocco L (2021). Protective effects of Curcumin on the outcome of Cryopreservation in Human sperm. Reprod Sci.

